# Inactivation of a purine biosynthesis repressor promotes ribosome synthesis to overcome antibiotic stress

**DOI:** 10.1128/mbio.00118-26

**Published:** 2026-04-13

**Authors:** Alexandre Le Scornet, Yongjun Tan, Dapeng Zhang, Jue D. Wang, Mee-Ngan F. Yap

**Affiliations:** 1 Department of Microbiology-Immunology, Northwestern University Feinberg School of Medicine 12244, Chicago, Illinois, USA; 2 Department of Biology, College of Arts and Sciences, Saint Louis University 169022, St. Louis, Missouri, USA; 3 Program of Bioinformatics and Computational Biology, Saint Louis University 7547, St. Louis, Missouri, USA; 4 Department of Bacteriology, University of Wisconsin-Madison 205263, Madison, Wisconsin, USA; Universite de Geneve, Geneva, Switzerland

**Keywords:** PurR, *Staphylococcus aureus*, antibiotic resistance, macrolide, ribosome, nucleotide metabolism, RNA methylation, erythromycin

## Abstract

Macrolide, lincosamide, and streptogramin (MLS) antibiotics are structurally distinct molecules that inhibit protein synthesis by binding overlapping sites within the 23S rRNA of bacterial ribosomes. The clinical utility of MLS antibiotics has diminished due to the dissemination of erythromycin resistance rRNA methyltransferase (Erm) genes. *Staphylococcus aureus* ErmB methylates the universally conserved A2058 nucleotide of the 23S rRNA (m^6^A2058), resulting in cross-resistance to all MLS antibiotics by reducing drug-binding affinity. The operonic upstream ribosome stalling peptide ErmBL was thought to be the sole regulatory element controlling ErmB synthesis and the extent of MLS resistance. Unexpectedly, our laboratory evolution experiments revealed that numerous loss-of-function mutations outside the bicistronic *ermBL-ermB* operon amplify ErmB-mediated MLS resistance. Among these are mutations in genes critical for purine *de novo* biosynthesis and the salvage pathway. Specifically, inactivation of the gene encoding the purine biosynthesis transcriptional repressor (PurR) converts an otherwise moderately resistant *ermBL-ermB (ermB^+^*) strain into one exhibiting pronounced hyper-resistance to MLS antibiotics. This cooperative resistance phenotype is not attributable to increased *ermB* expression or elevated m^6^A2058 modification. Instead, *purR* inactivation leads to derepression of purine and pyrimidine biosynthesis, accompanied by increased expression of ribosome components. We find that the elevated ribosome abundance and translational capacity of the *ermB^+^*∆*purR* are directly proportional to its accelerated growth rate, thereby priming *S. aureus* for survival under high MLS concentrations. These findings support a model in which expanded nucleotide metabolites and a surplus of antibiotic-free ribosomes drive global translation to buffer the inhibitory effects of MLS antibiotics.

**IMPORTANCE:**

The Erm rRNA methyltransferase superfamily represents the most prevalent determinant of MLS resistance in nosocomial Gram-negative and Gram-positive bacteria. Previous studies have established that *erm* expression is primarily governed by upstream ribosome stalling peptide and the 5′ untranslated regions. Using the widespread *S. aureus ermBL-ermB* (*ermB*^+^) operon as a model system, we unexpectedly identified second-site mutations in *purR* that synergistically enhance MLS resistance in an *ermB*^+^ background. Loss of *purR* function derepresses nucleotide biosynthesis and ribosome production, thereby promoting bacterial growth under antibiotic stress. While numerous *purR* single-nucleotide polymorphisms across multiple species have been associated with antibiotic resistance, no study has directly linked these sequence polymorphisms to their regulatory function. Our results highlight the critical role of ribosome abundance and nucleotide metabolism in shaping antibiotic efficacy.

## INTRODUCTION

Macrolide antibiotics are the third most consumed class of antibiotics globally and first-line treatments for nontuberculosis mycobacteria, *Bordetella pertussis*, and atypical bacterial pneumonia (1–4). They are also critical for treating *Staphylococcus aureus* infections in patients with penicillin allergy. Lincosamide and streptogramin antibiotics are chemically distinct protein synthesis inhibitors that share overlapping 23S rRNA binding sites in the bacterial ribosomes with macrolides. Mutations and modifications of the common binding sites confer cross-resistance to all macrolides, lincosamides, and streptogramins, collectively termed MLS resistance. The most pervasive mechanism of MLS resistance is dimethylation of the universally conserved A2058 nucleotide in 23S rRNA (m^6^A2058, *E. coli* numbering) by the Erm rRNA methyltransferase superfamily (5–10), which disrupts hydrogen bonding between the antibiotics and 23S rRNA and reduces drug-binding affinity (11, 12).

Members of the *erm* subfamilies are among the 37 Rank I high-risk antibiotic resistance gene families identified by the World Health Organization as “current threats” due to their high genetic mobility, enrichment in human-associated environments, and presence in all ESKAPE pathogens, including both Gram-negative and Gram-positive bacteria (5, 6, 10, 13, 14). Amplification of *erm*-containing plasmid via clonal spread of multiple sublineages of methicillin-resistant *S. aureus* (MRSA) has been reported (15). All 49 subfamilies of *erm* are invariably encoded downstream of a macrolide-inducible ribosome stalling leader sequence in a bicistronic operon (16, 17). The classical “translational attenuation” model proposes that subinhibitory concentrations of macrolide stall the translating ribosome within the *erm* leader sequence, which in turn rearranges the conformation of an inhibitory mRNA structure between the leader and *erm*. This conformational change unmasks the translation start site and/or Shine-Dalgarno (SD) sequence of the downstream *erm* gene, allowing translational initiation and upregulation of Erm synthesis (16). This paradigm has been challenged by observations that mutations in the *erm* leader peptide that impair ribosome stalling, as well as sequence variations in the 5′ untranslated region, are extremely prevalent in natural isolates compared with the model *erm* operons. These sequence polymorphisms often lead to constitutive overexpression of Erm (18–31). However, overexpression of Erm enzymes imposes a fitness loss *in vitro* and in a murine model of bacteremia, likely due to the translational inefficiency and infidelity of methylated ribosomes (32, 33).

*S. aureus* is the second leading cause of global mortality and morbidity associated with antimicrobial resistance and can infect nearly every human tissue and organ, including the blood, heart, and lungs (34–38). Nasal colonization of *S. aureus* has also been linked to depression (39). Using the MRSA *ermBL-ermB* operon as a model system, our previous machine-learning analyses of ~22,000 *ermB* upstream sequences demonstrated that mutations in *ermBL* and the stability of inhibitory intergenic mRNA structure largely determine ErmB expression levels and the extent of MLS resistance (29). Mutations within *ermB* itself can also enhance methyltransferase activity (12, 33, 40). Beyond sequence variations within *ermBL-ermB*, however, it remains unknown whether other genomic changes can impact *ermB* expression and MLS resistance. In this laboratory-directed evolution study, we discover that loss-of-function mutations in the *de novo* purine biosynthesis repressor PurR enhance ErmB-mediated MLS resistance without altering ErmB levels or m^6^A2058 methylation status. RNA-seq analysis reveals that deletion of *purR* strikingly upregulates purine and pyrimidine biosynthetic genes, as well as genes encoding ribosomal proteins and translation factors, concomitant with accelerated bacterial growth. This growth promotion is linked to increased global protein synthesis and ribosome production. Together, our results demonstrate that nucleotide metabolism and ribosome content can significantly alter antibiotic resistance spectrum, highlighting the need for better understanding the contribution of bacterial metabolic state to drug efficacy.

## RESULTS

### Loss-of-function *purR* mutations augment MLS resistance in the *erm*^+^ strain

Indels and nonsense mutations in *erm* leader regulatory regions are prevalent in natural isolates (18–29). These sequence polymorphisms often impair translation of the *erm* leader peptide and disrupt inhibitory mRNA structures, leading to constitutive expression of Erm and high-level MLS resistance. We previously found that *ermB* upstream sequence polymorphisms strongly correlate with the degree of MLS resistance and the expression levels of ErmB methyltransferase (29, 30). Strains carrying an intact *ermBL-ermB* operon are moderately resistant to MLS antibiotics. To determine whether genomic changes outside of the *ermBL-ermB* operon also contribute to MLS resistance, we experimentally evolved an *S. aureus ermBL-ermB* strain (hereinafter referred to as the *ermB^+^* strain) by serial passages in increasing concentrations of either erythromycin (ERY; a macrolide) or clindamycin (CLN, a lincosamide) for 14–18 days. This selection yielded ERY and CLN resistance levels of up to 2.3 mg/mL and 1 mg/mL, respectively, from initial minimum inhibitory concentrations (MIC) of 12 μg/mL and 0.38 μg/mL. The parental MLS-susceptible JE2 strain lacking *ermBL-ermB* was denoted as the *ermB*^−^ strain. Mutations arising during evolution were identified by whole-genome sequencing of endpoint mixed populations and compared against the ancestral *ermB****^+^*** strain that underwent identical serial transfers without antibiotic exposure. Variants were detected using a widely adopted C++-based breseq computational pipeline (41, 42), enabling identification of new junctions and single-nucleotide mutations at base pair resolution. Nonsynonymous mutations were found in functionally diverse genes involved in transcription, translation, and metabolism (Table 1; Data set S1). Two genes, *purR* and *apt*, were of particular interest because *purR* [GAA→TAA (E184*), GTA→GGA(V229G)], and *apt* [AAA→GAA(K82E), TTC→TGC(F20C)] mutations were repeatedly detected across multiple antibiotic-exposed lines. The purine biosynthesis transcriptional repressor (PurR) regulates *de novo* purine biosynthesis, whereas adenine phosphoribosyltransferase (Apt) participates in adenine salvaging. PurR contains an N-terminal helix-turn-helix DNA-binding domain that recognizes *pur* boxes located at the promoters of multiple operons, and a C-terminal phosphoribosyl-transferase (PRT) domain responsible for ligand binding (Fig. S1). In *B. subtilis*, the nucleotide precursor phosphoribosyl pyrophosphate (PRPP) binds to the PRT domain, preventing PurR-mediated repression, whereas competitive binding of (p)ppGpp enhances PurR-DNA binding and represses purine nucleotide synthesis (43). The nonsense mutation E184* and the missense V229G identified here map to the PRT domain, suggesting impaired ligand sensing.

**TABLE 1:** Representative extragenic mutations that enhance ErmB-mediated MLS resistance^*a*^

Genes	Annotation	Position/nucleotide change	Protein change
*purR*	*pur* operon repressor	582,257 (G→T) 532,393 (T→G)	E184* (GAA→TAA) V229G (GTA→GGA)
*apt*	Adenine phosphoribosyltransferase	1,743,838 (T→C) 1,743,023 (A→C)	K82E (AAA→GAA) F20C (TTC→TGC)
*rluB*	23S rRNA pseudouridine synthase B	1,598,377 (A→T)	L42* (TTA→TAA)
*rpoC*	RNA polymerase beta subunit	590,064 (G→T)	G266C (GGT→TGT)
*ecsA*	Putative ABC transporter	1,968,468 (C→A)	E178* (GAA→TAA)
*lip*	Triacylglycerol lipase precursor	2,831,375 (C→A)	G381* (GGA→TGA)
bioA	Adenosylmethionine-8-amino-7-oxononanoate transaminase	2,549,677 (C→T)	R189H (CGC→CAC)
*dnaJ*	Chaperone protein DnaJ	1,688,528 (C→T)	A250T (GCT→ACT)
*recF*	DNA replication and repair protein	5,010 (C→G)	P358A (CCT→GCT)
*pfk*	PfkB family carbohydrate kinase	2,150,261 (+C/760 nt)	D253 (frameshift)

^*a*^ Mutations were identified by whole-genome sequencing following laboratory evolution in the presence of increasing concentrations of either erythromycin (a macrolide) or clindamycin (a lincosamide). An asterisk indicates nonsense mutation. A complete list of nucleotide polymorphisms is provided in Data set S1.

**Fig 1:**
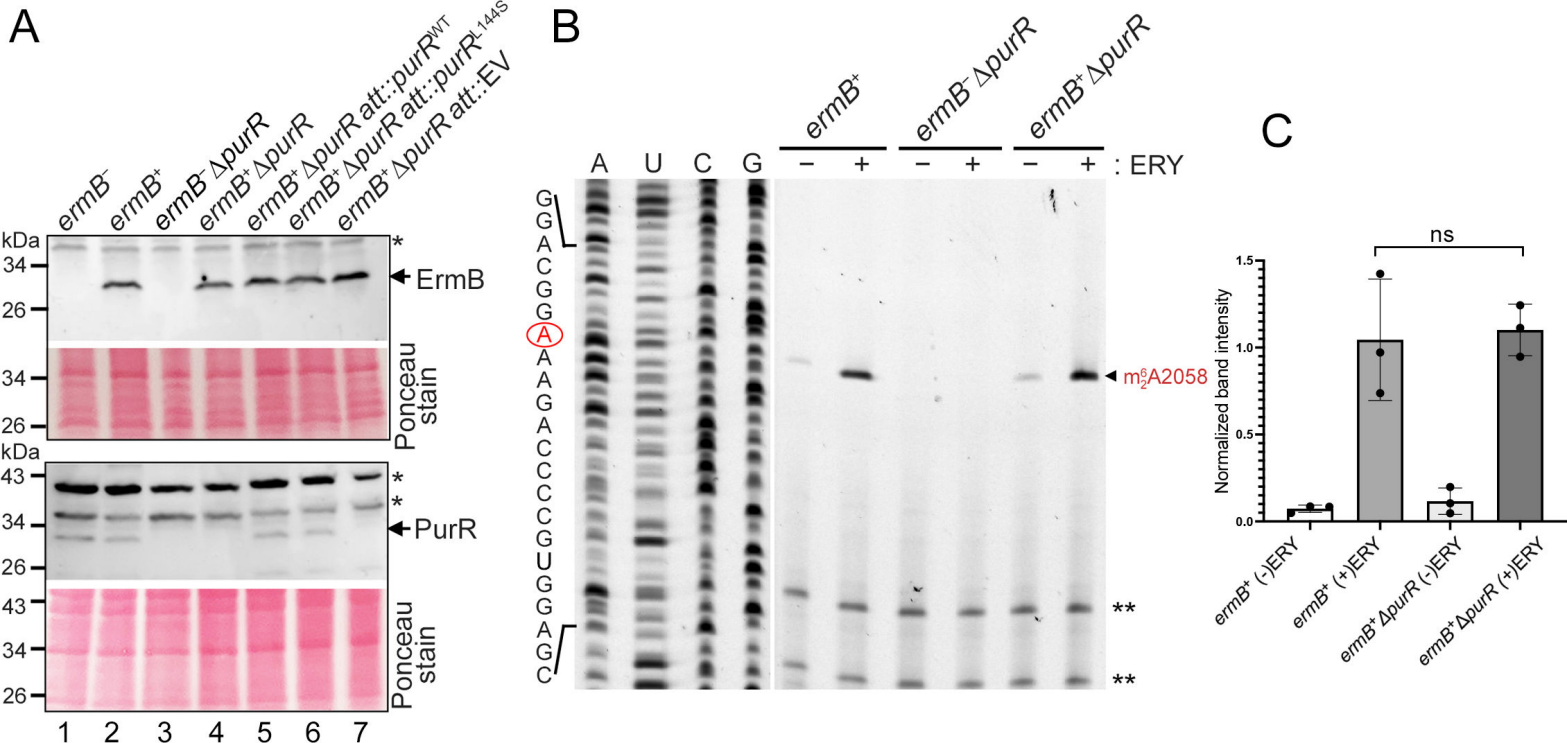
Inactivation of *purR* does not affect the expression of *ermB* or A2058 methylation levels. (**A**) Immunoblot analysis using anti-ErmB (1/500 dilution) and anti-PurR_Bs_ (1/500 dilution) antibodies from MHB cultures treated with a sublethal concentration of erythromycin (ERY; 1 µg/mL). An asterisk denotes a nonspecific cross-reaction. EV, empty vector. All complemented alleles were integrated at the chromosomal *geh-att* site. (**B**) Primer extension assay showing the degree of A2058 methylation in 23S rRNA. Reverse transcriptase stalls 1 nt downstream of the methylated A2058 site, producing a truncated cDNA detected on a denaturing PAGE gel. ** denotes an internal reference for equal rRNA input. (−) and (+) indicate the absence or presence of a 90-minute treatment with 1 µg/mL ERY to induce *ermB* expression. (**C**) Quantification of m^6^A2058 modification from panel **B** relative to the internal reference bands (**). Signal intensities were measured using ImageJ and normalized to the *ermB*^+^∆*purR* strain. Student’s *t*-test (*n* = 3); ns, not significant.

We constructed clean *purR* and *apt* deletions in the *ermB^+^* strain. The *erm*^+^∆*purR* strain fully recapitulated the evolved resistance phenotype, increasing MICs of macrolides (ERY and AZ), the lincosamide CLN, and the ketolide SOL by 3- to 680-fold relative to the *ermB^+^* strain (Table 2). Deletion of *purR* did not alter MICs for antibiotics targeting other cellular components, with the exception of ciprofloxacin, due to an unexplained collateral sensitivity associated with the *ermB*^+^ allele (33). MLS resistance remained dependent on the presence of *ermBL-ermB*, as MICs of the *ermB^−^* and *ermB*^−^∆*purR* strains were almost identically low (Table 2). Importantly, chromosomal complementation with wild-type (WT) *purR*, but not the loss-of-function L144S variant, at a neutral *att* site fully resensitized *ermB^+^*∆*purR* cells to MLS (44, 45), excluding off-target effects. Ectopic *purR* expression was comparable to native levels, averting artifacts from overexpression (Fig. 1A, lane 2 vs lane 5). The L144S mutant was selected because this naturally occurring variant is required for the transition from colonization to invasive phenotypes and for antibiotic resistance in *S. aureus* (46, 47), and unlike E184* and V229G, the L144S variant retained the WT levels of expression (Fig. 1A, lane 2 vs lane 6). Lastly, deletion of *apt* in the *ermB^+^* background also increased MLS resistance, validating the *in vitro* evolution results. Conversely, expression of WT *apt in trans* restored ERY sensitivity, whereas the Apt(K82E) variant failed to complement the phenotype (Fig. S2). Collectively, these findings indicate that disruption of both *de novo* purine synthesis and salvage pathways can substantially alter MLS resistance profiles.

**TABLE 2:** Minimum inhibitory concentrations (MIC, µg/mL) of various antibiotics against *S. aureus* strains with and without *purR* and inducible *ermB*^*d*^

Cellular targets	Abx^*a*^	*erm* ^−^	*erm*^−^∆*purR*	*erm* ^+^	*erm*^+^∆*purR*	*erm*^+^∆*purR, att::purR* (WT)^*b*^	*erm*^+^∆*purR, att::purR* (L144S)^*b*^
50S ribosomal subunit	ERY	0.19	0.19	12	≥256	12–16	≥256
	AZI	0.38	0.5	64	≥256	64	≥256
	CLN	0.032	0.047	0.38	≥256	0.38	≥192
	SOL	0.064	0.125	1.5	4	1.5	4
30S ribosomal subunit	KAN	1.5–2	1.5–2	1.5	1.5	1.5	1.5
	STP	0.38	0.38	0.38	0.38	nd^*c*^	nd^*c*^
Cell wall/membrane	DAP	1	1	1	1	1	1
	VAN	1.5	1.5	1.5	1.5	1.5	1.5
RNA polymerase	RIF	0.006	0.006	0.006	0.006	0.006	0.006
DNA gyrase	CIP	24	24	8-12	6-8	12	8

^*a*^ Abx, antibiotics; ERY, AZI (macrolides erythromycin and azithromycin), CLN (clindamycin, a lincosamide), SOL (solithromycin, a ketolide), Kan (kanamycin), STP (streptomycin), DAP (daptomycin), VAN (vancomycin), RIF (rifampicin), CIP (ciprofloxacin).

^*b*^ Single copy expressed PurR at the neutral chromosomal *geh_att* locus.

^*c*^ nd, not determined.

^*d*^ MICs were determined by E-test on Mueller-Hinton agar plates using 3–5 biological replicates per strain per antibiotic.

### Inactivation of *purR* does not alter *ermB* expression or the 23S rRNA methylation

Because deletion of *purR* additively enhanced *ermB-*mediated MLS resistance, we asked whether loss of *purR* increased *ermB* expression. However, Western blot analysis revealed no detectable difference in the steady-state ErmB protein levels between *ermB^+^* and *ermB*^+^∆*purR* strains (Fig. 1A, lane 2 vs lane 4). Primer extension assays further showed that the extent of m^6^A2058 methylation in the total RNA was nearly identical between *ermB^+^* and *ermB*^+^∆*purR* strains (Fig. 1B and C). rRNAs were also extracted from density gradient fractionated 70S complexes and analyzed by primer extension (Fig. S3A). The m^6^A2058 signals were indistinguishable between the *ermB^+^* and *ermB*^+^∆*purR* strains, ruling out that 23S rRNAs in mature ribosomes were differentially modified (Fig. S3B). These results exclude a direct regulatory role for PurR in controlling *ermB* expression or m^6^A2058 modification.

### Deletion of *purR* derepresses nucleotide biosynthesis and virulence genes and upregulates ribosomal protein genes

**Fig 2:**
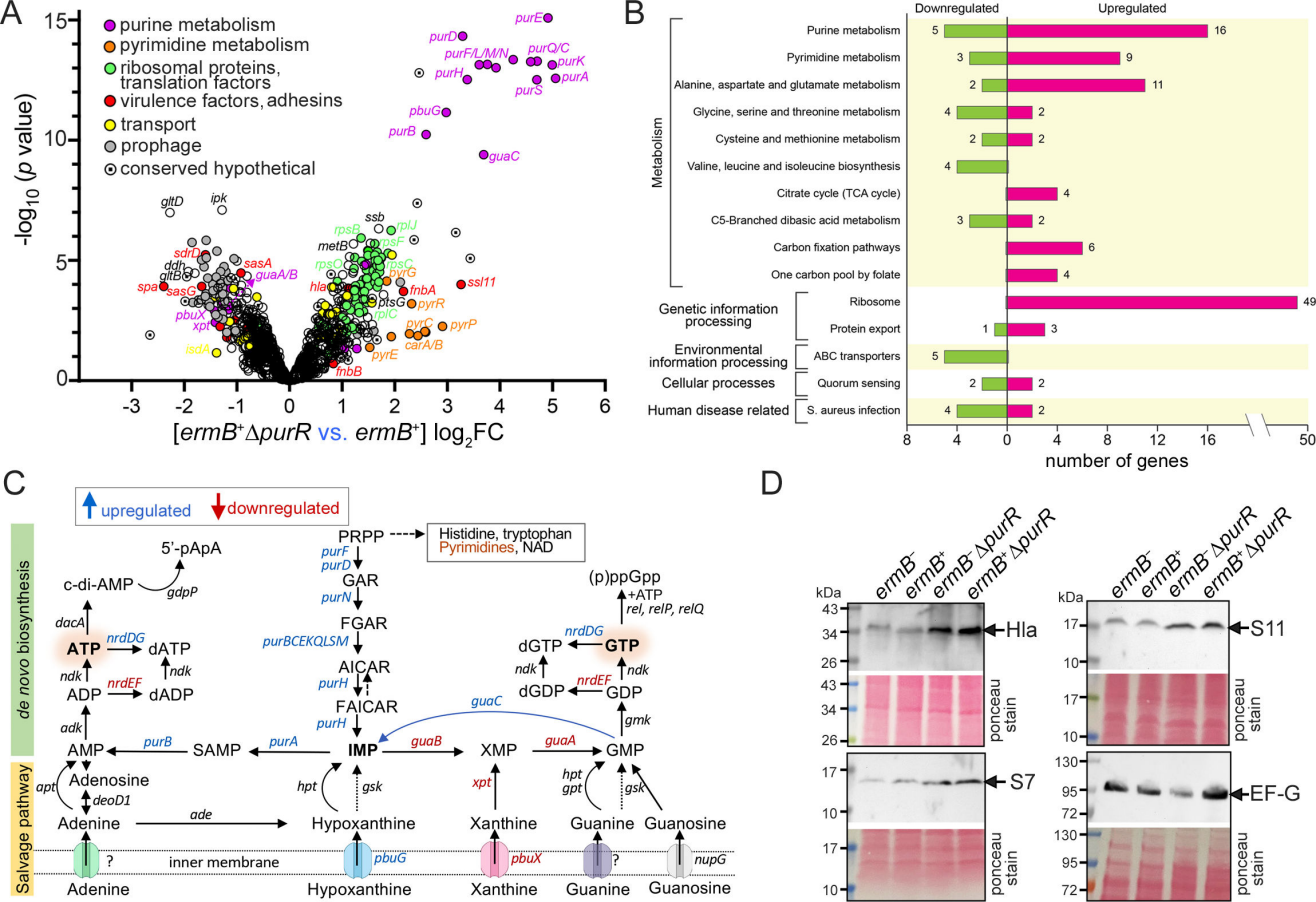
Genes encoding purine and pyrimidine biosynthesis pathways and ribosomal components are upregulated in ∆*purR* mutant backgrounds. (**A**) Volcano plot of differentially expressed genes showing ≥2-fold changes (*P* ≤ 0.05) between *ermB^+^*∆*purR* and *ermB*^−^ strains. A comprehensive comparison among four strains is provided in Data set S2. (**B**) KEGG pathway categorization of 147 genes from the 248 differentially expressed genes shown in panel **A**. Gene ontology (GO) analysis revealed enrichment in biological processes of translation and purine-pyrimidine metabolism. (**C**) Simplified illustration of *de novo* and salvage purine biosynthesis pathways in *S. aureus*. Blue and red indicate genes upregulated or downregulated, respectively, in the *ermB^+^*∆*purR* strain relative to the *ermB^+^* strain. (**D**) Validation of RNA-seq results by Western blotting. Increased levels of α-hemolysin (Hla), ribosomal proteins S7 and S11, and elongation factor G (EF-G) in the ∆*purR* background were confirmed.

Mutations in *purR* have been linked to increased resistance to antibiotics targeting diverse cellular processes in *S. aureus* (47–56), *S. pneumoniae* (57), and *E. coli* (58), although the underlying mechanisms remain poorly understood. We hypothesized that *purR* deletion alters gene expression programs that enhance MLS resistance. RNA-seq was performed to compare mRNA levels of *ermB*^−^, *ermB*^−^∆*purR*, *ermB^+^*, and *ermB*^+^∆*purR* strains during logarithmic growth (Data set S2). Consistent with the repressor function of PurR (59, 60), genes involved in purine and pyrimidine biosynthesis, virulence, and purine transport (*pbuG*, hypoxanthine permease) were strongly upregulated. In contrast, *guaA* (GMP synthase), *guaB* (IMP dehydrogenase), *xpt* (xanthine phosphoribosyltransferase), and *pbuX* (xanthine permease) were downregulated (Fig. 2A through C). PurR is known to directly repress key virulence genes in *S. aureus*, including superantigen-like protein 11 (*ssl11*), fibronectin-binding protein A (*fnbA*), and α-hemolysin (*hla*) (60), supporting our RNA-seq results. We validated selected targets by Western blotting, including Hla, ribosomal proteins S7 and S11, and elongation factor G (EF-G) (Fig. 2D). Although antibody availability limited validation breadth, elevated protein levels were consistent with corresponding mRNA upregulation following *purR* deletion.

Notably, 49 of 54 core ribosomal protein genes were significantly upregulated in the *ermB*^+^∆*purR* strain relative to the *ermB*^+^ strain (Fig. 2B), along with essential translation factors (EF-G, IF1, IF3) and ribosome biogenesis proteins such as RimM and TypA (Data set S2). The remaining five ribosomal proteins (bS1, bL9, uS14, bL12, bS21) are either dispensable, highly abundant, or can be functionally compensated by their paralogs (61, 62). Because ribosome abundance linearly correlates with growth rate under balanced exponential conditions (63, 64), these results suggest that *purR* deletion promotes cell growth, thereby enhancing MLS resistance.

### Deletion of *purR* accelerates cell growth

**Fig 3:**
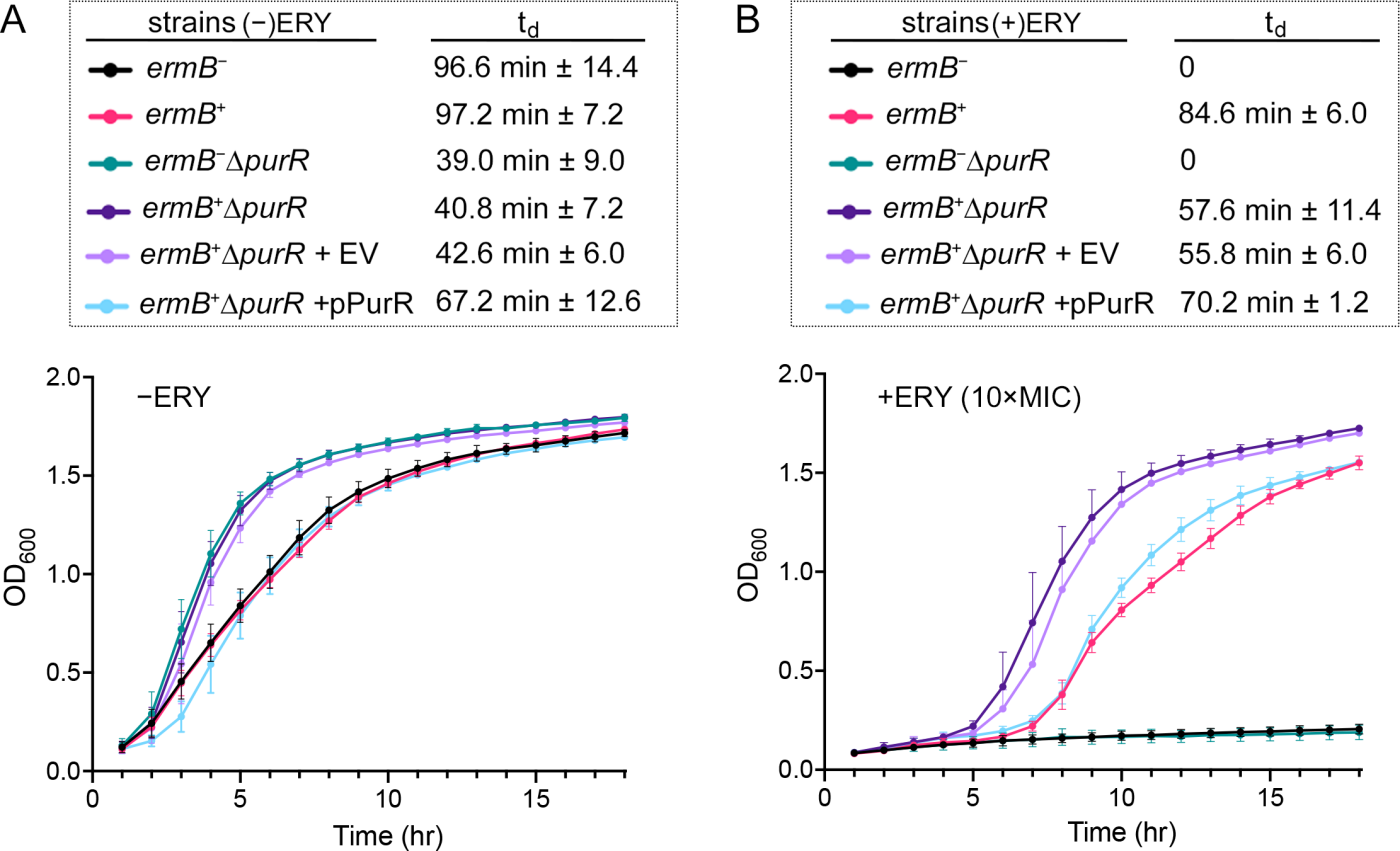
Growth kinetics and doubling times of *S. aureus* strains cultured in MHB in the absence (**A**) and presence (**B**) of 10× MIC erythromycin (ERY). Doubling time (*t*_*d*_) represents the mean ± SD from three independent experiments. EV, empty vector.

Reduced bacterial growth rates are often associated with increased antibiotic resistance (65, 66). We therefore measured growth kinetics of *purR* mutant derivatives in cation-adjusted MHB media, the standard medium for MIC determination. Contrary to expectation, the hyperresistant *ermB^+^*∆*purR* strain exhibited a markedly shorter doubling time than its parental *ermB^+^*, both in the absence and presence of lethal ERY concentrations (Fig. 3). Deletion of *purR* reduced the doubling times by approximately half in both *ermB*^−^ and *ermB^+^* backgrounds without ERY treatment, indicating that loss of *purR* generally promotes rapid growth and supports a positive relationship between growth rate and ribosome content. Finally, *in vitro* competition experiments between *ermB*^+^ and *ermB^+^*∆*purR* strains verified that the *ermB^+^*∆*purR* strain consistently exhibited a competitive advantage both in the absence and presence of subinhibitory ERY (Fig. S4). Overall, the introduction of the ∆*purR* allele enhances bacterial fitness *in vitro*.

The stringent response alarmone (p)ppGpp suppresses cell growth during nutritional limitation by inhibiting metabolic enzymes and macromolecule synthesis (67, 68), but its impact on antibiotic susceptibility varies across drug classes and species (69–71). In *S. aureus*, Rel, RelP, and RelQ catalyze the production of (p)ppGpp using ATP and GTP/GDP substrates, thereby depleting cellular GTP and ATP pools and slowing cell growth (67, 72). Given that *purR* deletion increases purine synthesis and MLS resistance, we postulated that a (p)ppGpp^0^ strain [strain lacking all (p)ppGpp synthetase domains] (73) would similarly affect antibiotic resistance in the *ermB*^+^ background. As hypothesized, the ∆*rel*∆*relP*∆*relQ-ermB*^+^ strain showed a modest increase in ERY resistance compared with the parental *ermB*^+^ strain, and combining the ∆*purR* allele with ∆*rel*∆*relP*∆*relQ-ermB*^+^ additively enhanced ERY resistance (Fig. S5), suggesting that PurR- and (p)ppGpp-stimulated resistance represent two independent pathways. These findings support the idea that perturbations of purine homeostasis can potentiate antibiotic resistance (74).

### The ∆*purR* mutant increases ribosome content and global protein synthesis

**Fig 4:**
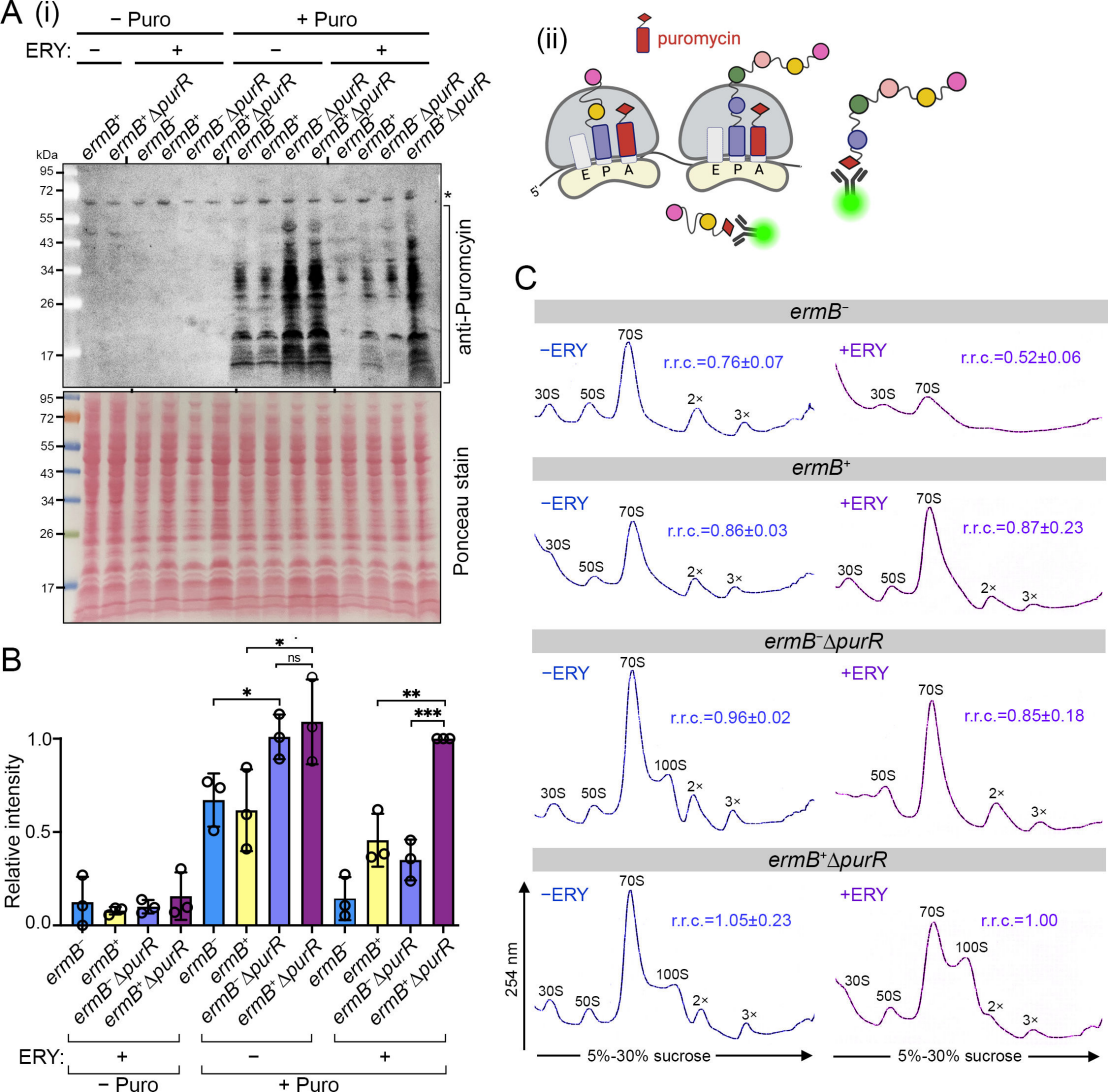
Inactivation of *purR* enhances global protein synthesis. (**A**) (i) Western blot-based SUnSET assay showing increased levels of fluorescently labeled puromycinated polypeptides in ∆*purR* backgrounds. (ii) Schematic illustrating puromycin (Puro) as an aminoacyl tRNA mimic that binds to the ribosomal A site and prematurely terminates elongation by transferring the growing nascent polypeptide. The resulting puromycylated peptides serve as a proxy for protein synthesis. (**B**) Densitometric quantification of panel **A**, normalized to the *ermB*^+^Δ*purR* lane using iBright analysis software. Data are shown as mean ± SD. Statistical significance was determined by one-way ANOVA with Dunnett’s multiple comparison test, ***P ≤ 0.001, **P ≤ 0.01, *P ≤ 0.05; ns, not significant. (**C**) Ribosome sedimentation profiles of *ermB^−/+^* and ∆*purR* strains in the presence and absence of 1 µg/mL erythromycin (ERY). Relative ribosomal content (r.r.c.; mean ± SD, *n* = 3) was calculated using the *ermB*^+^Δ*purR* strain as a reference (set to 1) by integrating areas under the ribosomal peaks with ImageJ, and total ribosomal areas were divided by that of an *ermB*^+^Δ*purR* strain. Panel ii was created using Biorender.

To link *purR* loss with elevated ribosome production (Fig. 2) and accelerated cell growth (Fig. 3), we quantified active translation using the SUnSET puromycin incorporation assay (75) coupled with Western blotting. Puromycin is a tyrosyl-tRNA mimic antibiotic that prematurely terminates elongation by entering the ribosomal A site and releasing nascent polypeptide. Fluorescently labeled anti-puromycin antibodies were used to detect newly synthesized proteins (Fig. 4A). Puromycin labeling was specific because puromycin-free cells exhibited minimal background signals. Consistent with growth kinetics (Fig. 3), *ermB^−^*∆*purR* and *ermB^+^*∆*purR* strains displayed elevated protein synthesis relative to their *ermB^−^* and *ermB^+^* counterparts in the absence of sublethal ERY. Upon ERY treatment, when *ermB* expression was induced, the *ermB^+^*∆*purR* strain exhibited the highest translational output (Fig. 4A and B).

The increased global translation could be due to upregulation of ribosomal protein genes (Fig. 2), leading to synthesis and assembly of translationally competent ribosomes. We next assessed ribosome abundance by sucrose density gradient ultracentrifugation to resolve 30S, 50S, 70S, 100S (70S dimer), and polysome fractions. Ribosome species were quantified by integrating areas under the curve using equivalent total ribosome input. The *purR*-proficient cells typically exhibit approximately 1:1:2–3 ratio of 30S:50S:70S ribosomal particles in the absence of ERY. Upon deletion of *purR*, this ratio increases to approximately 1:1:5–6 ratio, reflecting ribosome overproduction. The *ermB^+^*∆*purR* strain produced the largest ribosome pools both with and without ERY exposure, consistent with its elevated translational capacity (Fig. 4C). Strains lacking *ermB* (*ermB*^−^ or *ermB*^−^∆*purR*) were extremely sensitive to subinhibitory ERY, resulting in defects in ribosome biogenesis and a sharp reduction in 70S complexes (Fig. 4C). Of note, dormant 100S ribosomes were significantly enriched in the *ermB^+^*∆*purR* strain under both ERY-treated and untreated conditions, correlating with upregulation of *hpf* (Data set S2), which encodes the hibernation factor responsible for dimerizing 70S ribosomes to form 100S complex. In ERY-sensitive *ermB*^−^∆*purR* cells, ERY inhibits ribosome biogenesis, thereby depleting 70S precursors and preventing the formation of 100S ribosomes (Fig. 4C).

The accumulation of 100S ribosomes in ∆*purR* backgrounds likely reflects increased ribosome synthesis and subsequent sequestration of 70S ribosomes that are not actively engaged in mRNA translation by Hpf. In *S. aureus*, 100S ribosomes represent RNase-resistant, inactive particles poised for reactivation under favorable conditions (76–79). These dormant ribosomes can later dissociate into active subunits to support rapid translational reinitiation and cell proliferation. However, unlike other Hpf homologs (80), *S. aureus* Hpf has not been implicated in antibiotic resistance, and its expression is not altered in (p)ppGpp-deficient cells (81), arguing against a direct role for Hpf in MLS resistance.

## DISCUSSION

**Fig 5:**
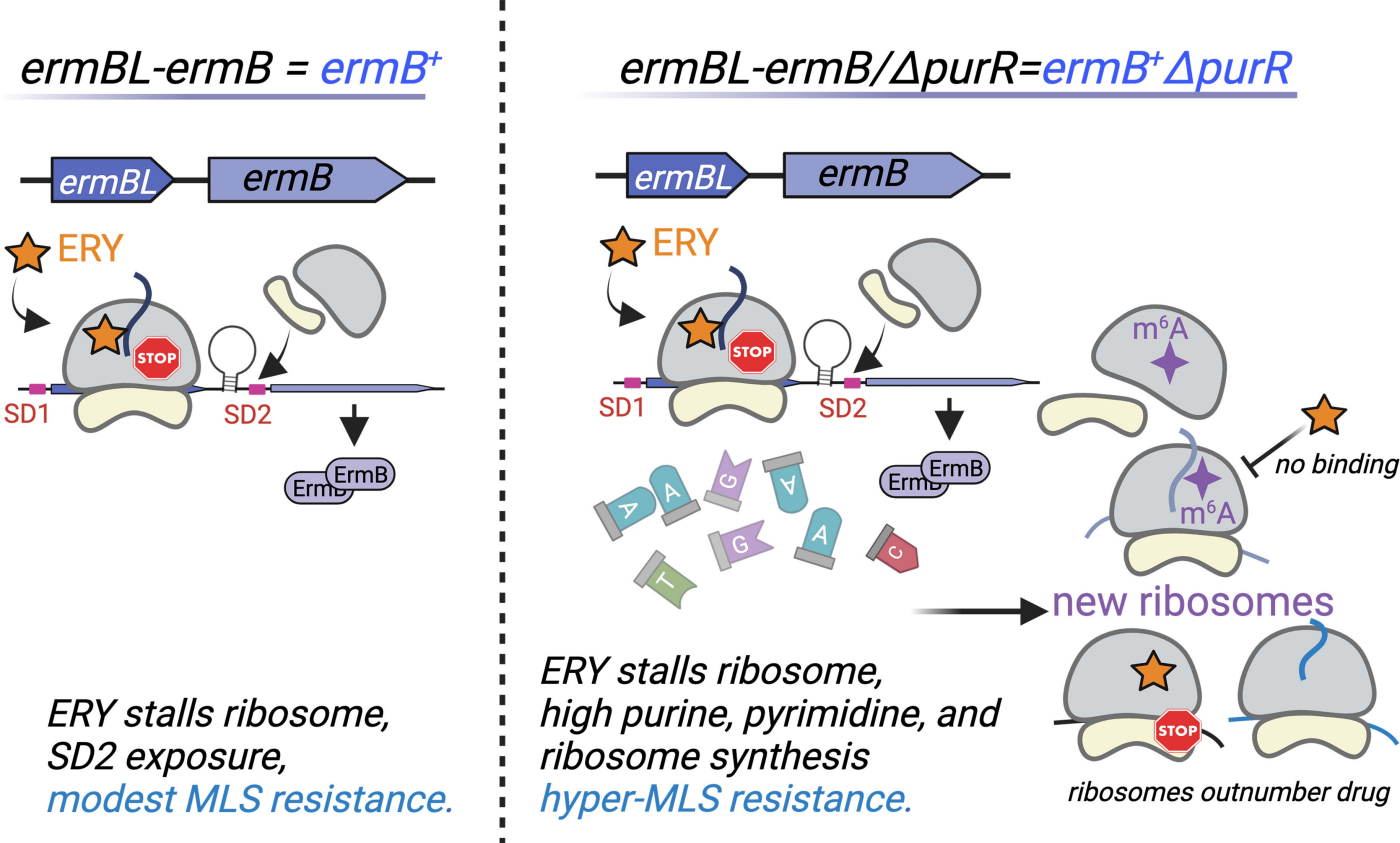
Proposed model for the role of PurR in modulating MLS resistance. (Left) Sublethal concentrations of erythromycin (ERY) arrest ribosome within the *ermBL* leader sequence, inducing mRNA structural rearrangements that expose the translation start site and/or Shine-Dalgarno 2 (SD2) to activate downstream *ermB* translation, resulting in modest ERY resistance. (Right) Loss of *purR* in the *ermB*^+^ strain elevates MLS resistance by expanding purine and pyrimidine nucleotide pools and derepressing ribosome synthesis. The resulting excess of methylated and unmethylated ribosomes promotes hyper-resistance by reducing antibiotic binding to modified ribosomes and by titrating antibiotic molecules through the expanded pool of unmodified ribosomes, thereby enabling sustained mRNA translation under antibiotic stress. Illustration created by Biorender.

Our *in vitro* evolution study identifies an unexpected set of second-site mutations that enhance ErmB-dependent MLS resistance, linking dysregulated nucleotide metabolism and ribosome biogenesis to amplification of antibiotic resistance. Loss of PurR not only derepresses genes involved in nucleotide synthesis and virulence but also upregulates nearly all ribosomal protein genes. Elevated nucleotide pools and increased ribosome biogenesis promote MLS resistance through two synergistic pathways: (i) enhanced purine and pyrimidine synthesis supplies precursors for DNA and RNA, and ATP and GTP to fuel ribosome assembly and translation; and (ii) excess unmethylated ribosomes titrate MLS antibiotics away from inhibiting translation, whereas m^6^A2058-ribosomes resist MLS binding (Fig. 5). Disruption of the adenine salvage pathway (∆*apt*) and the stringent response (ppGpp^0^) also increases MLS resistance (Fig. 2C; Fig. S2 and S5), though less than that of ∆*purR*, and the underlying mechanisms remain to be determined.

The modest increase in erythromycin resistance observed in the *ermB*^+^ppGpp^0^ strain may appear paradoxical, given that disruption of the stringent response in other species often leads to increased antibiotic sensitivity (69, 71, 82). A key distinction between our findings and previous studies is the presence of the inducible-resistance determinant *ermB*. Strains lacking *ermB* but carrying the *ppGpp^0^* allele (*ermB*^−^ ppGpp^0^ or ∆*purR* ppGpp^0^) remain highly susceptible to erythromycin and are slightly more sensitive than the parental *ermB*^−^ strain (Fig. S5). Furthermore, in *S. aureus*, (p)ppGpp does not regulate transcription through direct binding to RNA polymerase. Instead, these alarmones influence gene expression indirectly by reducing intracellular GTP levels and by serving as an effector of PurR (43, 67). Consequently, the impact of the stringent response on antibiotic susceptibility is highly context-dependent, influenced by factors such as bacterial species [e.g., whether (p)ppGpp directly interacts with RNA polymerase], the class of antibiotic (bacteriostatic vs bactericidal), the initiating nucleotide of transcription (see below), the preexisting resistance determinants, and the growth environment.

Genomic surveillance of patient isolates and laboratory-evolved *S. aureus* reveals a high prevalence of nonsynonymous mutations and indels in *purR* that affect antibiotic susceptibility and virulence (Fig. S1A) (46–49, 51, 55, 56, 59, 83–87). Mutations in *apt* have also been associated with resistance to nafcillin, monensin, and vancomycin (56, 88–90). These mutations influence diverse antibiotic classes, ranging from rifampicin, daptomycin, silver, vancomycin, nisin, moenomycin A, to monensin. However, most mutations have not been validated by strain reconstruction and complementation, and no unifying mechanism of PurR-mediated antibiotic resistance has emerged. Notably, the E184* and V229 mutations identified here have been detected in hypervirulent *S. aureus* (59), and in strains resistant to nisin (which targets lipid II) (48) and DNA gyrase inhibitors (55). Resistance-associated *purR* mutations also occur in *S. pneumoniae* and *E. coli* isolates (57, 58). The prevalence of *purR* mutations and their association with resistance to antibiotics targeting distinct cellular sites underscore the pleiotropic effects of *purR* disruption and suggest involvement of core cellular processes such as nucleotide metabolism and protein synthesis.

The *S. aureus* PurR DNA-binding motif has been loosely mapped to a degenerate AT-rich sequence (60). Genes carrying PurR box include the *purEKCSQLFMNHD* operon, *purA*, *purB*, the *fnbAB* operon, *ssl11*, and the ribosomal *rpsF-ssb-rpsR*, *rplM-rpsI*, and *rpmF* operons identified in previous studies and in our RNA-seq (60). Our computational scanning of PurR binding motif, derived from RegPrecise database (91), using MEME program (92), identified additional PurR boxes upstream of the *pbuG*, *hlgABC*, *opuCabcd*, and *rrnA* operons (Data set S3; Fig. S6A). Upregulation of *pbuG*, *hlgB*, *hlgC*, and *opuCabcd* is also detected as part of the PurR regulon in our RNA-seq analysis (Data set S2). The presence of a PurR box upstream of the *rrnA* operon is significant because it encodes the 16S, 23S, and 5S rRNAs, which are integral components of the ribosomes. These rRNAs were not detected in our RNA-seq analysis due to the rRNA depletion prior to library preparation. The detection of the PurR-binding motif in the *rrnA* operon and operons encoding ribosomal proteins reinforces the notion that increased ribosome content in the ∆*purR* mutant contributes to its hyper-resistance to MLS. Unlike *rrn* operons in *E. coli*, which are regulated by (p)ppGpp binding to RNA polymerase, (p)ppGpp in Firmicutes, including *S. aureus,* does not directly interact with RNA polymerase. Instead, transcriptional reprogramming during the stringent response occurs indirectly through a reduction in GTP levels (67). As a result, promoters that use GTP as the initiating nucleotide (+1 to +4 positions) are particularly sensitive to fluctuations in GTP concentration. The two transcriptional start sites of the *S. aureus rrnA* operon have been mapped to GTP-initiating nucleotides (Fig. S6A) (93), and *rrnA* transcription is therefore repressed under stringent response. Our work suggests that removal of *purR* not only increases ATP and GTP pools and derepresses the *rrnA* promoter, but also eliminates the (p)ppGpp target PurR, collectively promoting the synthesis of *rrnA*-encoded rRNAs. Variations in bacterial rRNA can affect gene expression (94, 95). *S. aureus* USA300 carries five *rrn* operons, and single-nucleotide polymorphisms (SNPs) in the PurR-regulated *rrnA* operon could potentially influence MLS resistance (Fig. S6B). However, none of the five SNPs map to the reported MLS resistance-associated mutations (6), ruling out a direct contribution of these SNPs.

Loss-of-function *purR* mutations contribute to a hypervirulence phenotype due to upregulation of genes required for host invasion and immune evasion (59, 60, 96). This phenotype is uncoupled from purine biosynthesis, as deletion of purine biosynthetic genes still increases bacterial burden in mice (60, 96), further suggesting that overproduction of purines is not the only contributing factor to virulence enhancement in the *purR* mutant. We initially hypothesized that MLS susceptibility of the *ermB*^+^ strain resulted from purine deficiency and that *purR* deletion would restore resistance by replenishing purine pools. However, exogenous purine and pyrimidine supplementation did not alter MLS susceptibility (not shown), indicating that purine overproduction alone is insufficient to boost resistance. Instead, our data suggest that elevated ribosome synthesis is required to counteract MLS stress (Fig. 5). Attempts to reverse MLS resistance by deleting purine biosynthetic genes (*purA*, *purF*, and *purM*) in the *ermB*^+^∆*purR* strain caused severe growth defects (not shown), precluding conclusive interpretation of MLS susceptibility.

Bacterial growth rates are directly proportional to the number of ribosomes in a cell (97). The observation that fast-growing cells are less susceptible to antibiotic treatment has been described (98–100). In one case, isogenic fast-growing *E. coli* strains that overproduce ribosomes and thus express more efflux pumps are more resistant to antibiotics. However, our RNA-seq analysis does not detect changes in efflux gene expression. Therefore, it is possible that increasing ribosome content is sufficient to buffer the inhibitory effects of ribosome-targeting antibiotics.

The global burden of antimicrobial resistance has created a demand to better understand the basic mechanisms of existing antibiotics, particularly how bacterial metabolism influences drug efficacy and how metabolic adaptability boosts antibiotic resistance (87, 101, 102). Prior work and our findings suggest that *purR* mutations may generate pathogen subpopulations primed for subsequent evolution of target-specific antibiotic resistance alleles. Small-molecule ligands, such as (p)ppGpp analogs, that enhance PurR DNA-binding activity may therefore serve as antibiotic adjuvants to inhibit growth while suppressing virulence and evolution of resistance.

## MATERIALS AND METHODS

### Bacterial strains, plasmid, chemicals, and growth conditions

The strains and plasmids used in this study are listed in Table S1. Oligonucleotides were purchased from IDT DNA (Table S2). Strain JE2 is a methicillin-resistant *Staphylococcus aureus* (MRSA) of USA300 lineage, cured of all three native plasmids. JE2 strain carrying the *ermBL-ermB* operon at the neutral chromosomal *att* site (strain KES29, *ermB*^+^) was constructed previously (33, 103). The C-terminally FLAG-tagged *ermBL-ermB*-3×FLAG allele was created by incorporating 3×FLAG sequence into pJC1111-*ermBL-ermB* plasmid (33) using the Gibson Assembly Kit (New England Biolabs). Spectinomycin resistance-marked ∆*purR* (MNY221) was constructed by allelic exchange of pSpc (104) with ∆*purR::erm* (strain NR-47780). QuikChange site-directed mutagenesis (Agilent Genomics) or Gibson Assembly was used to introduce point mutations. Various mutant alleles were subsequently transferred to the KES29 or JE2 derivatives by Φ11 phage transduction.

The in-frame *apt* deletion strain was created using standard allelic-exchange recombination using the temperature-sensitive pBT2 vector (105, 106), following previously described procedures (107). Single-copy *purR* complements were constructed using the pCL55 suicide vector that integrates the *purR* alleles into the *geh-att* site of the chromosome (44, 45). Complemented strains of ∆*apt* were made by expressing *apt* alleles on the xylose-inducible pEPSA5 using Gibson Assembly Kit (NEB) (108). Unless otherwise noted, *S. aureus* cells were grown aerobically at 37°C in tryptic soy broth (TSB; BD Difco) or cation-adjusted Mueller-Hinton Broth (MHB; BD Difco) at a 5:1 tube- or flask-to-medium ratio. *E. coli* cells were grown in LB (BD Difco). When necessary, antibiotics were supplemented at the following final concentrations: erythromycin (1 µg/mL to 400 µg/ml), chloramphenicol (10 µg/mL), kanamycin (100 µg/mL), spectinomycin (1 mg/mL), cadmium chloride (0.15 mM), and ampicillin (100 µg/mL). All chemicals were obtained from Sigma-Aldrich unless otherwise noted.

### Experimental evolution of macrolide and clindamycin resistance

The evolution experiments were performed in 4 mL MHB cultures of *S. aureus* KES29 with seven (clindamycin) and five (erythromycin) replicate lines evolving for each antibiotic. The initial concentrations of clindamycin and erythromycin were 1.5 µg/mL and 12 µg/mL, corresponding to 3× MIC and 1× MIC of KES29 (*ermB*^+^), respectively (Table 1). Cultures were incubated at 37°C for 20 h, after which 4 µL of each culture was transferred into 4 mL of fresh MHB (1: 1,000 dilution) containing antibiotic concentrations increased by 0.5× relative to the previous passage. In parallel, five replicate KES29 populations were serially passaged in antibiotic-free MHB to serve as controls for estimating the basal mutation rate over the same experimental time frame. Freezer stocks were prepared every third passage, beginning at passage 3. After 14-18 days of serial passaging, clindamycin-evolved and erythromycin-evolved populations reached resistance levels of 980 µg/ml and 1,548 µg/mL, respectively. Genomic DNA was immediately extracted from 2 mL of the final evolved populations using the Quick-DNA Fungal/Bacterial Kit (Zymo Research), according to the manufacturer’s protocol. DNA library preparation and whole-genome sequencing were performed by MiGS (now SeqCoast) on an Illumina platform with 150 bp paired-end reads. Sequencing fastq files were analyzed and aligned to the reference genome (GenBank CP000255) as well as to the ancestral KES29 strain passaged without antibiotic selection. Point mutations and indels were identified using the breseq pipeline (version 0.33.0) (41, 42) with default parameters for SNP calling.

### Measurement of minimum inhibitory concentration (MIC)

MIC values of various antibiotics were determined by antibiotic E-test strip diffusion assays (Biomerieux or Liofilchem) on the cation-adjusted MHA (BD Difco) plates following the manufacturer’s instruction. MICs were recorded after 24-hour incubation at 37°C.

### Western blotting

Total *S. aureus* lysates from MHB or TSB cultures were extracted with Lysing Matrix B in 25 mM Tris-HCl (pH 7.5) on a FastPrep-24 (MP Biomedicals) homogenizer as described previously (78). A total of 0.1–0.2 *A*_280_ units of cell lysate were resolved on 4–20% TGX SDS-PAGE (Bio-Rad), transferred to a nitrocellulose membrane, followed by immunoblotting using anti-ErmB (1/500 dilution) (43), anti-PurR_BS_ (1/500 dilution) (43), anti-S7 (1/1,000 dilution) (79), anti-S11 (1/2,000 dilution) (77, 109), anti-EF-G (1/ 7,000 dilution) (76), or anti-Hla (1/1,000 dilution, IBT BioServices). For anti-ErmB production, N-terminally His_6_-tagged ErmB was expressed on pET28a in *E. coli* BL2(DE3) and purified using Ni-NTA affinity column (MCLAB) under native conditions. Polyclonal anti-ErmB was raised in rabbits (Josman, LLC). Anti-ErmB sera were preabsorbed with JE2 lysate before hybridization to minimize nonspecific cross-reaction.

### RNA-seq

Overnight cultures were diluted 1/100 into fresh TSB or RPMI 1640 plus 1% casamino acids and grown at 37°C. When cultures reached OD_600_ of 0.2, cells were treated with erythromycin at final concentrations of 1 µg/mL or 400 µg/mL for 180 min. Untreated control cultures were harvested at an OD_600_ of 0.8. For each condition, 20 mL of culture was collected and resuspended in 1 mL T_10_E_1_ buffer. Cells were disrupted using a FastPrep24 homogenizer (MP Biomedicals) for 1 min with Lysing Matrix B beads. Cellular debris was removed by centrifugation, and total RNA was extracted twice with acidic phenol/chloroform (pH 4.5), followed by a single extraction with chloroform-isoamyl alcohol (24:1). Total RNA was further cleaned up using Qiagen RNeasy Mini columns and treated with TURBO DNA-free DNase I to remove residual genomic DNA. RNA quality was assessed on a Bioanalyzer (Agilent Genomics). Illumina sequencing library construction and sequencing were performed by MiGS (now SeqCoast) and the NuSeq Core (Northwestern University) using the Illumina Stranded Total RNA Prep Ligation Kit with Ribo-Zero Plus and Illumina Unique Dual Indexes.

Sequencing quality control and adapter trimming were performed using bcl2fastq (Illumina). Reads were mapped to the reference genome using HISAT2 (110). Gene-level read counts were generated using the featureCounts function in Subread (111). Read counts were imported into R (112) and were normalized using the trimmed mean of M values (TMM) algorithm implemented in edgeR (113). Subsequent normalized values were converted to counts per million (cpm). Differential expression analysis was conducted using edgeR’s exact test for pairwise comparisons of negative binomially distributed counts, with a dispersion value set to 0.1. Gene Ontology (GO) analysis using Blast2GO (114) and Kyoto Encyclopedia of Genes and Genomes (KEGG) pathway enrichment analysis (115) were used to classify differentially expressed genes.

### Measurement of bacterial doubling time

*S. aureus* strains were grown at 37°C in MHB media, and optical density OD_600_ was monitored on a Tecan SPARK microplate reader equipped with a humidity chamber. Erythromycin was added to a final 100 µg/mL when needed. Doubling times were calculated using GraphPad Prism 10 by fitting growth curves to a nonlinear regression model and deriving the growth rate constant (*k*). The doubling time *t*_*d*_ was calculated using the equation *t*_*d*_ = ln(2)/*k*, based on the steepest exponential growth phase within the OD_600_ range of 0.1–0.9.

### Primer extension to detect m^6^A2058 status

Overnight cultures of *S. aureus* strains were diluted 1/100 in fresh TSB or MHB and treated with erythromycin 1 µg/mL at OD_600_ = 0.3 for 90 min. Ten milliliters of culture was harvested, and pellets were resuspended in 1 mL T_10_E_1_. Total RNA was isolated as described in “RNA-seq.” rRNA was isolated from the 70S ribosomal fraction (see “Ribosome sedimentation”) by phenol-chloroform (pH 4.5) extraction as previously described (78). A total of 250 ng of RNA was used to carry out reverse transcription using fluorescently labeled primer 5′-(6-FAM)-TCCTGTACAACGTGTGCCGAAT (Table S2), in reactions containing SuperScript III Reverse Transcriptase (Thermo Fisher). The resulting cDNA was extracted with phenol/chloroform (pH 6.8) and precipitated with isopropanol. DNA sequencing ladders were generated using a USB Thermo SEQ Kit (Affymetrix) and *S. aureus* 23S rDNA (PCR amplified and column purified) as a template. Samples were heat-denatured and resolved on a 10% TBE/urea PAGE sequencing gel. Sequencing gel was scanned using a Typhoon 5 Imager (Cytiva).

### Surface sensing of translation (SUnSET) assay

A Western blot version of SUnSET (75) was used to measure *de novo* protein synthesis in *S. aureus*. Briefly, *S. aureus* strains were grown in MHB at 37°C with shaking at 200 rpm to an OD_600_ of 1.0. Cultures were split into equal aliquots and treated with or without 1 µg/mL erythromycin, in the absence or presence of 100 µg/mL puromycin (32× MIC), followed by an additional 90-minute incubation. Cytoplasmic proteins were extracted using a FastPrep 24 homogenizer and quantified by Bradford assay. Approximately 20 µg of total protein per sample was resolved on 4–20% SDS-PAGE gel, transferred onto nitrocellulose membranes, and immunoblotted with AlexaFluor conjugated anti-puromycin antibody (Sigma-Aldrich #MABE3430AF488) at a 1/1,000 dilution. Images were acquired using the iBright FL1500 system (Thermo Fisher). Relative protein synthesis levels were quantified using iBright Analysis Software by integrating fluorescence signals across the full molecular weight range of puromycin-incorporated polypeptides in each lane. Ponceau S staining of the membrane was used to confirm comparable protein loading across all samples.

### Ribosome sedimentation profiles

Crude ribosomes were isolated from *S. aureus* by cryo-milling methods in buffer A (20 mM HEPES [pH 7.5], 14 mM magnesium acetate, 100 mM KCl, 0.5 mM phenylmethylsulfonyl fluoride, and 1 mM dithiothreitol) as previously described (116). Ribosome extracts corresponding to five absorbance units at 260 nm (*A*_260_) were layered onto 5%–30% sucrose gradient prepared using a BioComp Gradient Master. Gradients were centrifuged at 210,000 × *g* at 4°C in an SW41 rotor in a Beckman Coulter Optima XPN-100 ultracentrifuge for 3 h. Fractionation was performed using a Brandel fractionation system equipped with a UA-6 UV detector. To quantify the total ribosome abundance relative to the *ermB^+^*Δ*purR* strain, ribosomal peak boundaries were manually defined at the troughs between adjacent peaks. Areas under the peaks were calculated using ImageJ, and relative ribosome content (r.r.c.) was determined by normalizing each sample to the ERY-treated *ermB^+^*Δ*purR* strain.

## Data Availability

The RNA-seq data reported in this paper have been deposited in the NCBI Gene Expression Omnibus (GEO) database with accession numbers GSE298918 and GSE299333. All other relevant data are within the article and its supplemental material.

## SUPPLEMENTAL MATERIAL

Data set S1 mbio.00118-26-s0001.xlsx Site mutations in *ermB*+ strain after erythromycin and clindamycin challenges.

Data set S2 mbio.00118-26-s0002.xlsx Differentially expressed genes in the presence or absence of *purR* and/or *ermB*+.

Data set S3 mbio.00118-26-s0003.xlsx Detection of PurR box-like motif in differentially expressed genes identified from RNAseq analysis.

Supplemental material mbio.00118-26-s0004.pdf Captions for Data sets S1 to S3; Tables S1 to S2; Fig. S1 to S6.

ASM does not own the copyrights to Supplemental Material that may be linked to, or accessed through, an article. The authors have granted ASM a non-exclusive, world-wide license to publish the Supplemental Material files. Please contact the corresponding author directly for reuse.
