# Supplementary material for: Inactivation of a purine biosynthesis repressor promotes ribosome synthesis to overcome antibiotic stress
Source: mBio. 2026 Apr 13;17(5):e00118-26. doi: 10.1128/mbio.00118-26 (PMC13170274; doi:10.1128/mbio.00118-26)
Supplement: Supplemental material — Captions for Data sets S1 to S3; Tables S1 to S2; Fig. S1 to S6. [file mbio.00118-26-s0004.pdf]

SUPPLEMENTAL MATERIALS

Inactivation of a purine biosynthesis repressor promotes ribosome synthesis to overcome antibiotic stress

Table S1. Bacterial strains and plasmids

Table S2. Oligonucleotides used in this study

Figure S1- Figure S6

Dataset S1. A full list of second-site mutations in *ermB*$^{+}$ strain after erythromycin and clindamycin challenges.

Dataset S2. Differentially expressed genes in the presence or absence of *purR* and/or *ermB*$^{+}$ in *S. aureus* strains grown in TSB (A) or RPMI 1640+1% casamino acids (B).

Dataset S3. Detection of PurR box-like motif in differentially expressed genes identified from RNA-seq analysis. Gray shaded rows indicate purine and pyrimidine synthesis genes, and genes encoding ribosomal constituents.

Supplementary References

Table S1. Bacterial strains and plasmids.

Strains	Genotypes	Antibiotic marker(s)	Reference
S. aureus USA300 JE2	Plasmid cured LAC strain (*ermB*$^{-}$)	−	BEI resources (1)
CM05	*ermB, cfr, mec*	multidrug	BEI resources (1)
RN4220	*sau1*$^{-}$, *hsdR*$^{-}$, *mec*$^{-}$, *rsbU*$^{-}$, *agr*$^{-}$, plasmid passage host	−	BEI resources (1)
KES29	JE2 *att:: ermBL-ermB* (*ermB*$^{+}$)	$CdCl_2$	(2)
NR-47780	JE2 Δ*purR*::Erm	Ery	BEI resources (1)
MNY221	JE2 Δ*purR*::Spc	Spc	This work
MNY224	KES29 Δ*purR*::Spc	$CdCl_2$, Spc	This work
MNY354	MNY224 *geh-att:*pCL55 empty	$CdCl_2$, Spc, Cm	This work
MNY337	MNY224 *geh-att:p*PurR^{WT}	$CdCl_2$, Spc, Cm	This work
MNY338	MNY224 *geh-att:p*PurR^{L144S}	$CdCl_2$, Spc, Cm	This work
MNY258	Δ*apt::*Km	Km	This work
MNY262	Δ*apt::*Km, *att:: ermBL-ermB* (*ermB*$^{+}$)	Km	This work
ALSY169	MNY262(pEPSA5)	Km	This work
ALSY148	MNY262(pEPSA5-apt^{WT})	Km, Cm	This work
ALSY296	MNY262(pEPSA5-apt^{K82E})	Km, Cm	This work
USA300_229_230_269 (ppGpp^{0})	JE2 Δ*rel*(Δ249-951), Δ*relP*(Δ450-536), Δ*relQ* (Δ343-429)	−	(3)
ALSY77	JE2 *ermBL-ermB*-3×FLAG	$CdCl_2$	This work
ALSY97	JE2 *ermBL-ermB*-3×FLAG, Δ*purR*::Spc	$CdCl_2$, Spc	This work
MNY275	JE2 *ermBL-ermB*-3×FLAG, ppGpp^{0}	$CdCl_2$	This work
ALSY196	JE2 ppGpp^{0}, Δ*purR*::Spc	Spc	This work
ALSY198	JE2 *ermBL-ermB*-3×FLAG, ppGpp^{0}, Δ*purR*::Spc	$CdCl_2$, Spc	This work
E. coli			
DC10B	DH10B Δ*dcm*, cloning host for *S. aureus* plasmids	−	(4)
**Plasmids**			
pEPSA5	*E. coli-S. aureus* shuttle vector, pT5X xylose-inducible promoter	Amp (*E. coli*) Cm (*S. aureus*)	(5)
pBT2	Temperature-sensitive *E. coli-S. aureus* shuttle vector	Amp (*E. coli*) Cm (*S. aureus*)	(6)
pBTK	Temperature-sensitive *E. coli-S. aureus* shuttle vector, 1.4-kb *aph-A3* cloned into *SmaI* site of pBT2.	Amp (*E. coli*) Cm, Km (*S. aureus*)	(7)
pJC1111	SaPI1 *attS* suicide vector	Amp (*E. coli*) $CdCl_2$(*S. aureus*)	(8)
pEPSA5-apt^{WT}	*S. aureus apt*^{WT} cloned into pEPSA5.	Amp (*E. coli*) Cm (*S. aureus*)	This work

pEPSA5-apt^{K82E}	*S. aureus apt*^{K82E} cloned into pEPSA5.	Amp (*E. coli*) Cm (*S. aureus*)	This work
pCL55	*geh-att* single-copy integrative plasmid	Amp (*E. coli*), Cm (*S. aureus*)	(9)
pCL55- $pPurR^{WT}$	*purR*^{WT}carrying its native promoter on pCL55.	Amp (*E. coli*), Cm (*S. aureus*)	This work
pCL55- $pPurR^{L144S}$	*purR*^{L144S} carrying its native promoter on pCL55.	Amp (*E. coli*), Cm (*S. aureus*)	This work
pLI50	promoterless *E. coli*-*S. aureus* shuttle vector	Amp (*E. coli*), Cm (*S. aureus*)	(9)
pLI50-$pPurR^{WT}$	*purR*^{WT}carrying its native promoter on pLI50	Amp (*E. coli*), Cm (*S. aureus*)	This work
pLI50-$pPurR^{L144S}$	*purR*^{L144S} carrying its native promoter on pLI50	Amp (*E. coli*), Cm (*S. aureus*)	This work
pET28a	IPTG-inducible overexpression vector	Km	Novagen
pET28a-ermB	*ermB* of *S. aureus* CM05 cloned into the NdeI and XhoI sites of pET28a. N-terminally His-tagged.	Km	This work

Table S2. Oligonucleotides used in this study

Primer	Sequence (5'-3')[a]	Application
P1723(SacI)f<br>P1724 (SmaI)r<br><br>P1725(SmaI)f<br><br>P1726 (BamHI)r	TTGGAGCTCCGAAAATTGTCGAAACATTTGCTTTGC<br>TGCCGCGTACTCTGCGCCCGGGATATCCATTAATATTT CCTCCTAAATT<br>ATCCCGGGCGCAGAGTACGCGGCATAATAAATAATATA ATTTTATCAAATG<br>TAGGATCCAACCCAAGTATCGCATAACAATCATTA	Crossover PCR to in-frame delete *apt* coding region and cloned into the SacI and BamHI sites of pBT2.
P1727f<br>P1728r	AGCAAAAGAAAATTGGCATGAAGGTGTCT<br>GGTTCTTATACATTTGATGAAGGATT	Δ*apt::*Km genotyping (WT~1.8-kb, Δ*apt::*Km ~2.8-kb)
P1447f<br>P1448r	CGAAATTGGAACAGGTAAAGGG<br>CTGACGATAAGTTGAATAGATGACTG	Δ*purR::*Spc Genotyping (WT~0.6-kb, Δ*purR::*Spc ~2.7-kb)
P2117(KpnI)<br>P2118(BamHI)	AGAGGTACCTCATCACGAATCAATTGCACACTAAA<br>TAAAGGATCCATAATACTAAAACTCCTTTTATGAAAAC	Cloning of P_{purR}-*purR* into pCL55
P1186f<br>P1187r	ATCTGCAGTTGGTCTTGCGTATGGTTAACCCTAAAG<br>TAGGTACCTAGAATTATTTCCTCCCGTTAAATAATAGA	Genotyping *ermBL-ermB* (~1.1-kb)
P1841f<br>P1842r<br>P1843f<br><br>P1844r	CTCTAGAGTCGACCTGCAGC<br>CCCGGGTACCGAGCTCGAA<br>AGCTCGGTACCCGGGGCAATTTAGGAGGAAATATTAAT GGATT<br>AGGTCGACTCTAGAGTTATTCGTCGTATGAGATTAAACT CATAACAT	Gibson assembly primers to construct pEPSA5::apt
P1805f<br>P1806r	AAAAGGTATTTCATCGGCAAATCGGGTTG<br>CAACCGCATTTGCCGATGAAATACCTTTT	Site-directed mutagenesis of PurR(L144S)
P2048f<br>P2048r	AATTACCTCGTGAAGTCATTC<br>CCCCTTCTTTTCTAACAGG	Apt(K82E) mutagenesis oligos
P2066f<br>P2067r	GTTGTTTTGTACATGGATTTTTAG<br>GCGCATAGGTGAGTTATTAGC	Confirm pCL55 integration into the chromosomal *geh-att* site (1.5-kb)
P1682r<br><br>P1679r	GATTATAAAGATCATGATATTGATTATAAAGATGATGAT GATAAATAAGGTACCGAGCTCGAATTCAG<br>ATGATCTTTATAATCGCCATCATGATCTTTATAATCTGA ACCTTTCCTCCCGTTAAATAATAGA	Primers to create *ermB*3×FLAG on pJC1111_*ermBL-ermB*.
P1430f<br>P1431r	GATGAGCTGTACAAATACTGCAGACCAGCGCG<br>CGCGCTGGTCTGCAGTATTTGTACAGCTCATC	pEPSA5 sequencing primers.
P2068f<br>P2069r	AATAGGCGTATCACGAGGC<br>TGATATTCAAGCCATTCATC	pCL55 sequencing primers.
P630f<br>P631r	GCACATTTCCCCGAAAAGTGCCACCTGACG T<br>TGCCTTTATTTTGAATTTTAAGGGGCA T	pLI50 sequencing primers.
FAM_Sa2058 _R2 (P1614)[b]	5'[6-FAM]-TCCTGTACAAGCTGTGCCGAAT	Primer extension to map m^6_2A2058

[a]: restriction enzyme cleavage sites are underlined. [b]: 6-FAM, 6-carboxyfluorescein

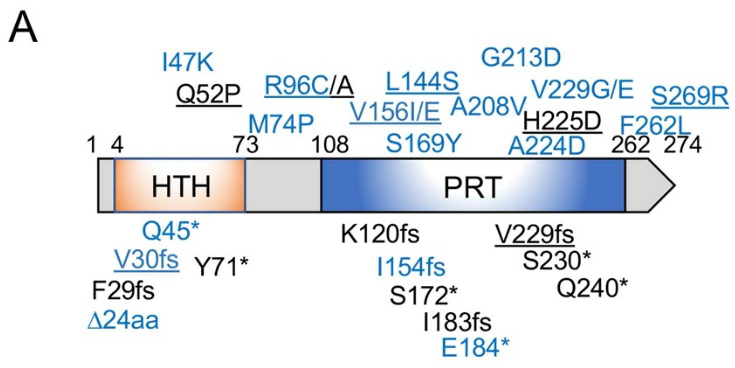

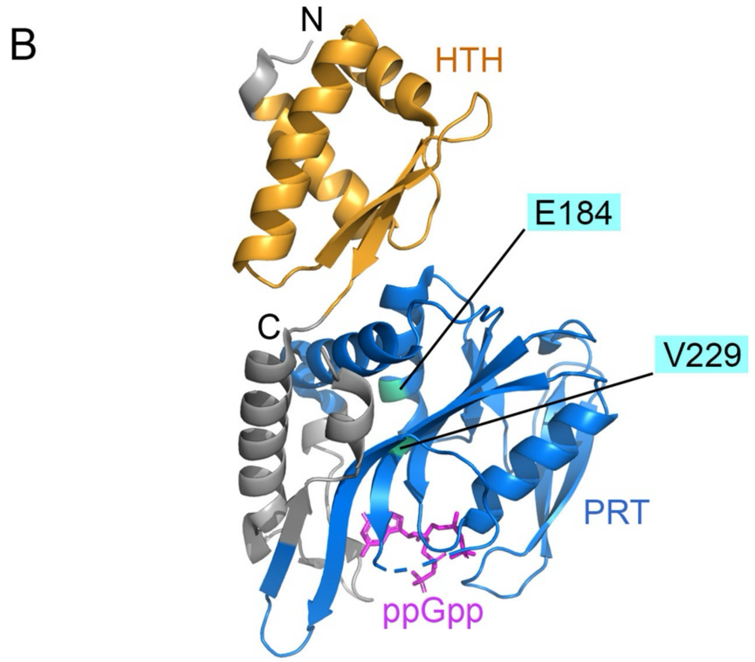

Figure S1. Domain architecture of PurR and SNPs identified in clinical isolates and laboratory evolution. (A) Distribution of PurR variants associated with antibiotic resistance (blue) and hypervirulence (underlined) in *S. aureus*. HTH, helix-turn-helix DNA-binding domain; PRT, phosphoribosyltransferase domain; fs, frameshift mutations; ∆, deletion. An asterisk indicates nonsense mutation. **(B)** Structural model of *S. aureus* PurR in complex with ppGpp (magenta) based on the published *B. subtilis* PurR structure (PDB 7rmw). E184* and V229G in the PRT domain are the two mutations identified in the *in vitro* evolution experiments.

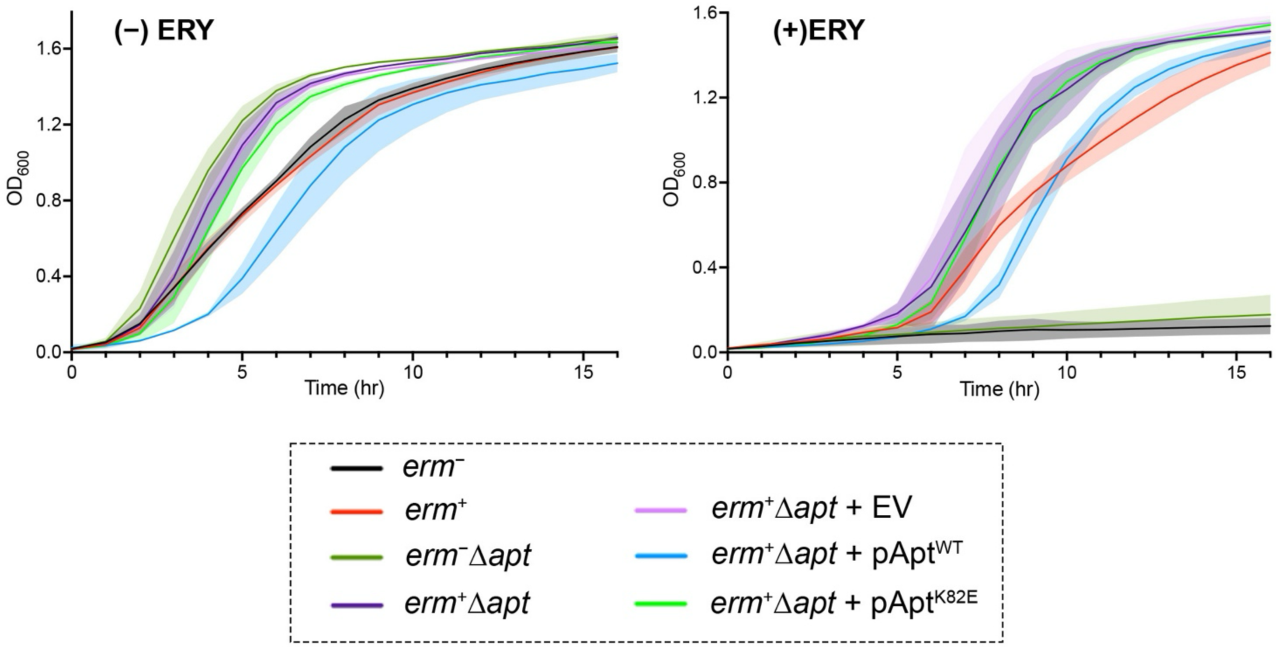

Figure S2. Complementation of the *ermB*⁺Δ*apt* resistance phenotype. Growth kinetics of *S. aureus* in MHB supplemented with or without 100 μg/ml erythromycin (ERY). Expression of *apt in trans* sensitizes the *ermB*⁺Δ*apt* strain to erythromycin. Growth kinetics were used as readout because Δ*apt* mutants form microcolonies and haze zones on solid MHB agar plates by an unknown mechanism, complicating agar-based antibiotic strip diffusion assays. Color-coded traces represent mean OD_{600}, and shaded regions indicate SD from three independent biological replicates. EV, empty pEPSA5 vector.

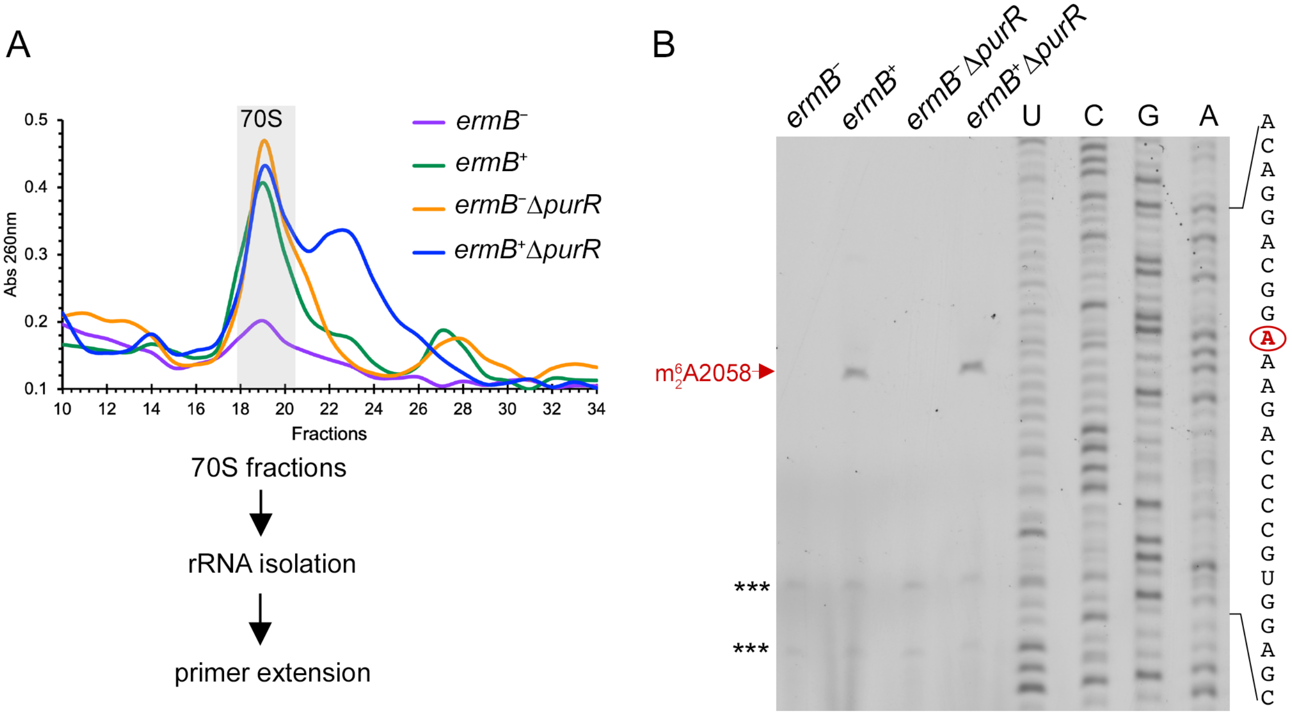

Figure S3. Inactivation of *purR* does not affect m^{6}A2058 levels in the 23S rRNA of mature 70S ribosomes. (A) Sucrose gradient (5%-30%) fractionation profiles of cell lysates prepared from *ermB*$^{-/+}$ and Δ*purR* strains treated with 1 μg/ml erythromycin (ERY) under identical MHB culture conditions as in Fig. 4C. **(B)** Primer extension analyses showing the extent of m^{6}A2058 methylation in 23S rRNA from the 70S ribosomes. *** denotes an internal reference band used to normalize rRNA input (250 ng). Representative image from two biological replicates.

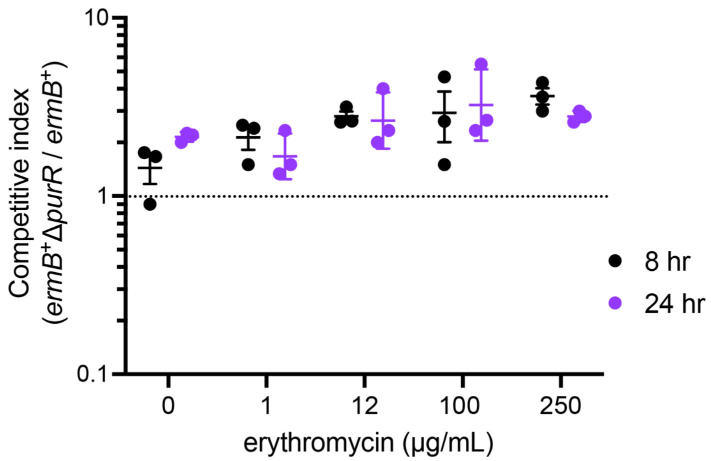

$$\text{Competitive index C. I} = \frac{\text{output}\left(\frac{ermB^{+}\Delta purR}{ermB^{+}}\right)}{\text{input}\left(\frac{ermB^{+}\Delta purR}{ermB^{+}}\right)}$$

Figure S4. *S. aureus ermB*$^{+}$ strain is outcompeted by the *ermB*$^{+}$Δ*purR* strain *in vitro* with and without antibiotic pressure. Bacterial cultures of *ermB*$^{+}$ and *ermB*$^{+}$Δ*purR* strains were adjusted to OD_{600}=0.2 (~10^9 CFU) and mixed at a 1:1 ratio in cation-adjusted MHB supplemented with 0, 1, 12, 100, 250 µg/mL erythromycin. Bacterial mixtures were plated onto TSB agar containing either 0.1 mM $CdCl_2$ (permissive for growth of both *ermB*$^{+}$ and *ermB*$^{+}$Δ*purR* strains) or 1 mg/mL spectinomycin (permissive only for the *ermB*$^{+}$Δ*purR* strain) before incubation and after 8 hr (black circles) and 24 hr (purple circles) of shaking at 37°C, 250 rpm. The competitive index (C.I.) was calculated as the ratio of output to input. C.I. > 1 indicates a competitive advantage of the *ermB*$^{+}$Δ*purR* strain.

Strains	ERY MIC (µg/mL)
ermB$^-$	0.19
ermB$^-$ ppGpp^0	0.125
ermB$^+$-FLAG	6
ermB$^+$-FLAG Δ*purR*	32-64
ermB$^+$-FLAG ppGpp^0	32
ppGpp^0 Δ*purR*	0.125
ermB$^+$-FLAG Δ*purR* ppGpp^0	96

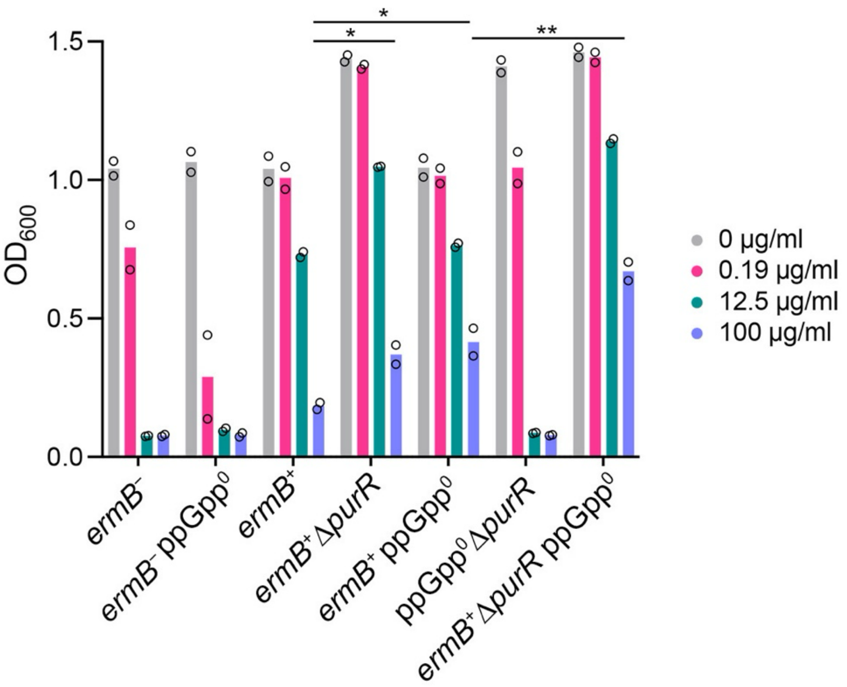

Figure S5. Elimination of the stringent response alarmone (p)ppGpp modestly enhances erythromycin (ERY) resistance. (Top panel) MICs of ppGpp^0 derivatives determined by E-test on Mueller Hinton agar plates in three biological replicates. (Bottom panel) Growth of *S. aureus* cultured in MHB containing increasing concentrations of ERY. Optical density OD_{600nm} was measured at the late logarithmic phase (~ 9 hr). Statistical significance was assessed using Student's t test, * $p \leq 0.05$; ** ≤ 0.01.

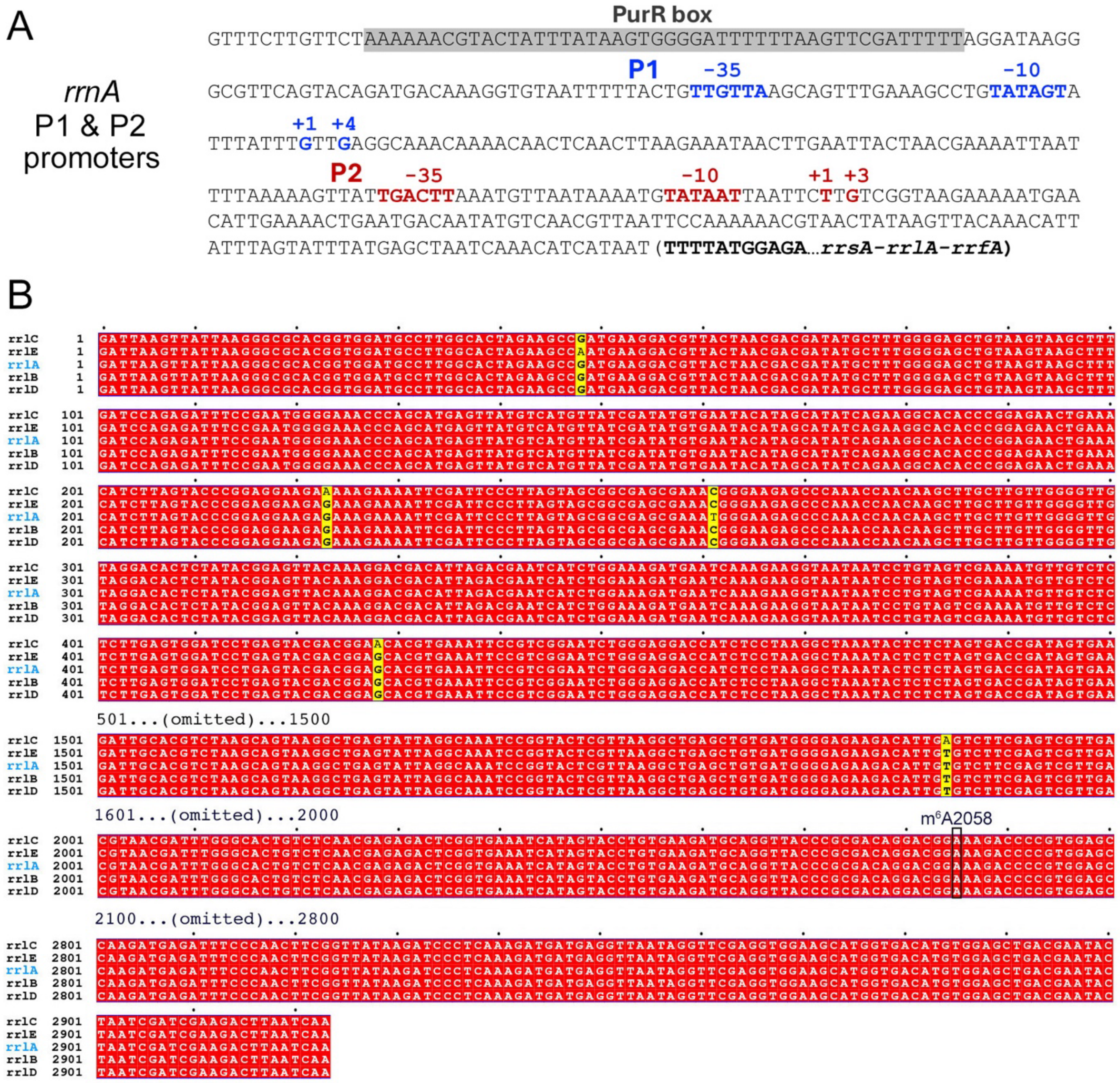

Figure S6. *S. aureus rrnA* promoter architecture and sequence variations among 23S rRNA-encoding *rrlA-rrlE* genes. (A) Schematic representation of the *rrnA* P1 and P2 promoters. Nucleotide positions are labeled relative to the transcriptional start site (+1). The -10 and -35 elements are highlighted in red or blue. The putative PurR-binding motif is indicated by a grey box. **(B)** Multiple sequence alignment of 23S rDNA from the five *S. aureus rrn* operons (*rrnA-rrnE*). Mutations in bacterial 23S rRNA that confer MLS resistance have been mapped to G748, A752, G2057, A2058, A2059, A2062, G2576 and C2611 (*E. coli* numbering). The *rrlA* gene (23S rDNA) from the *rrnA* operon contains five SNPs (yellow), none are located within known MLS resistance-associated regions. ErmB-methylated A2058 residue is indicated.

Supplementary References

1. Fey PD, Endres JL, Yajjala VK, Widhelm TJ, Boissy RJ, Bose JL, Bayles KW. 2013. A genetic resource for rapid and comprehensive phenotype screening of nonessential *Staphylococcus aureus* genes. mBio 4:e00537-00512.
2. Shields KE, Ranava D, Tan Y, Zhang D, Yap MF. 2024. Epitranscriptional m^6A modification of rRNA negatively impacts translation and host colonization in *Staphylococcus aureus*. PLoS Pathog 20:e1011968. PMCID: PMC10833563.
3. Horvatek P, Salzer A, Hanna AMF, Gratani FL, Keinhörster D, Korn N, Borisova M, Mayer C, Rejman D, Mäder U, Wolz C. 2020. Inducible expression of (pp)pGpp synthetases in *Staphylococcus aureus* is associated with activation of stress response genes. PLoS Genet 16:e1009282.
4. Monk IR, Shah IM, Xu M, Tan MW, Foster TJ. 2012. Transforming the untransformable: application of direct transformation to manipulate genetically *Staphylococcus aureus* and *Staphylococcus epidermidis*. mBio 3:e00277-00211.
5. Forsyth RA, Haselbeck RJ, Ohlsen KL, Yamamoto RT, Xu H, Trawick JD, Wall D, Wang L, Brown-Driver V, Froelich JM, C KG, King P, McCarthy M, Malone C, Misiner B, Robbins D, Tan Z, Zhu Zy ZY, Carr G, Mosca DA, Zamudio C, Foulkes JG, Zyskind JW. 2002. A genome-wide strategy for the identification of essential genes in *Staphylococcus aureus*. Mol Microbiol 43:1387-1400.
6. Bruckner R. 1992. A series of shuttle vectors for *Bacillus subtilis* and *Escherichia coli*. Gene 122:187-192.
7. Fuller JR, Vitko NP, Perkowski EF, Scott E, Khatri D, Spontak JS, Thurlow LR, Richardson AR. 2011. Identification of a lactate-quinone oxidoreductase in *Staphylococcus aureus* that is essential for virulence. Front Cell Infect Microbiol 1:19.
8. Chen J, Yoong P, Ram G, Torres VJ, Novick RP. 2014. Single-copy vectors for integration at the SaPI1 attachment site for *Staphylococcus aureus*. Plasmid 76:1-7.
9. Lee CY, Buranen SL, Ye ZH. 1991. Construction of single-copy integration vectors for *Staphylococcus aureus*. Gene 103:101-105.
